# Monoclonal gammopathy-associated C3 glomerulonephritis secondary to follicular lymphoma: a case report

**DOI:** 10.3389/fimmu.2025.1551788

**Published:** 2025-04-24

**Authors:** Wenjing Cai, Hanguo Guo, Minghui Zhang, Huaban Liang, Xinling Liang, Yihan Wang, Qingying Shi, Zhiming Ye, Zhilian Li

**Affiliations:** ^1^ Department of Nephrology, Guangdong Provincial People’s Hospital (Guangdong Academy of Medical Sciences), Southern Medical University, Guangzhou, Guangdong, China; ^2^ Department of Nephrology, Heyuan People’s Hospital, Guangdong Provincial People’s Hospital Heyuan Hospital), Heyuan, Guangdong, China; ^3^ Department of Lymphoma, Guangdong Provincial People’s Hospital (Guangdong Academy of Medical Sciences), Southern Medical University, Guangzhou, Guangdong, China; ^4^ Department of Pathology, Guangdong Provincial People’s Hospital (Guangdong Academy of Medical Sciences), Southern Medical University, Guangzhou, Guangdong, China; ^5^ Department of Nephrology, Guangdong-Hong Kong Joint Laboratory on Immunological and Genetic Kidney Diseases, Guangzhou, Guangdong, China; ^6^ Department of Pathology, Microbiology and Immunology, Vanderbilt University Medical Center, Nashville, TN, United States

**Keywords:** monoclonal gammopathy, C3 glomerulonephritis, follicular lymphoma, lymphoma-directed therapy, complement dysregulation

## Abstract

C3 glomerulopathy encompasses a group of glomerular diseases characterized by the predominant deposition of complement component C3 on kidney biopsy without significant immunoglobulin staining. Monoclonal gammopathy (MIg)-associated C3 glomerulopathy is considered a distinct subtype. We report a case of a 38-year-old male with a history of HBV infection who presented with a left neck mass, hematuria, proteinuria, elevated creatinine, normal complement level, and an IgM kappa M-spike on serum immunofixation electrophoresis. He was diagnosed with follicular lymphoma-associated MIg-C3 glomerulonephritis (MIg-C3GN), accompanied by extensive infiltration of lymphoma cells in the renal interstitium. Kidney immunohistochemistry was positive for CD19 and CD20 (B-cell markers), as well as CD10 and Bcl2, confirming the follicular lymphoma subtype. Within the areas of lymphoma cell infiltration, immunohistochemistry was also positive for IgM and kappa light chains but negative for lambda light chains, consistent with the serum electrophoresis findings. Positive autoantibodies to complement C3 convertase and complement factor H indicated complement dysregulation. The patient successively underwent various chemotherapy and targeted therapy regimens. During nearly 2 years of follow-up, renal outcomes were favorable, with resolution of hematuria and proteinuria and normalization of renal function, suggesting that lymphoma-directed therapy can improve renal outcomes in MIg-C3GN. However, the patient achieved partial remission but later relapsed and progressed, with suspicion of transformation into diffuse large B-cell lymphoma.

## 1 Introduction

C3 glomerulopathy comprises a group of glomerular diseases characterized by predominant complement component C3 deposition on kidney biopsy without evidence of significant immunoglobulin staining. Based on electron microscopy, C3 glomerulopathy is classified as two histologic patterns: dense deposit disease (DDD) and C3 glomerulonephritis (C3GN) (1). C3GN is characterized by mesangial and capillary wall electron dense deposits, whereas DDD is characterized by dense osmiophilic intramembranous and mesangial deposits. Recently, there has been increased recognition of the association between C3GN and monoclonal gammopathies. Monoclonal gammopathy-associated C3GN (MIg-C3GN) represents a distinct subgroup of diseases marked by abnormal clonal proliferation of Ig-producing B-lymphocytes or plasma cells (2). Lymphoma-associated C3GN is rare. Here, we present a case of a 38-year-old male with a history of hepatitis B virus infection, who was diagnosed with follicular lymphoma, exhibited an M-spike (monoclonal immunoglobulin in serum immunofixation electrophoresis), microscopic hematuria, mild proteinuria, acute kidney injury, and biopsy-proven C3GN with infiltrating lymphoma cells.

## 2 Case presentation

A 38-year-old Chinese male was referred to our hospital’s lymphoma department in March 2023 and subsequently to the nephrology department in April 2023, presenting with mild bilateral lower extremity edema, microscopic hematuria, proteinuria, elevated serum creatinine, and recurrent low-grade fever. His medical history included chronic hepatitis B virus (HBV) infection managed with lamivudine. In September 2022, while in Thailand, the patient noticed a left-sided neck mass but delayed medical consultant until late 2022, by which time he had lost 6 kg. A systemic PET/CT scan revealed multiple enlarged lymph nodes, hepatosplenomegaly and bilateral pleural effusion, with no initial signs of renal involvement. A biopsy of the left axillary lymph node and bone marrow aspiration confirmed a diagnosis of follicular lymphoma (grade 3A, stage IV A), with marrow involvement. Chemotherapy was initiated in Thailand with one cycle of R-COP (Rituximab, Cyclophosphamide, Vincristine, Prednisone) followed by two cycles of R-CHOP (Rituximab, Cyclophosphamide, Doxorubicin, Vincristine, Prednisone). Unfortunately, detailed records of his renal function, urinalysis, or immunoglobulin levels were not available during this period.

In March 2023, upon returning to China, the patient underwent a comprehensive evaluation in the lymphoma department, including lymph node biopsy, bone marrow aspiration biopsy, immunohistochemistry, *in situ* hybridization, FISH, and PET/CT. The PET/CT results showed multiple enlarged lymph nodes throughout the body with varying degrees of increased glucose metabolism, suggesting lymphoma with infiltration in the aforementioned areas, and no abnormalities were found in the kidneys. According to the 2023 version of the National Comprehensive Cancer Network (NCCN) lymphoma guidelines, the patient was diagnosed with follicular lymphoma (grade 3A, stage IV A) (3). During this period, his laboratory tests revealed a serum creatinine level of 131 μmol/L (normal range: 59-104 μmol/L), serum albumin of 38.3 g/L, a spot urine protein-to-creatinine ratio of 781 mg/gCr, and an albumin-to-creatinine ratio of 101 mg/gCr, indicating impaired kidney function and significant proteinuria. The lymphoma department prescribed one cycle of G-CHOP (Obinutuzumab, Cyclophosphamide, Doxorubicin, Vincristine, Prednisone) chemotherapy. However, within two days, the patient developed gross hematuria, accompanied by urinary urgency and pain, with significant presence of white and red blood cells in his urine. Although treatment with levofloxacin alleviated the hematuria, his serum creatinine increased to 218 μmol/L. Consequently, the patient was referred to the nephrology department.

Serum protein electrophoresis/immunofixation showed a monoclonal IgM kappa M-spike and an elevated IgM of 7.47g/L (normal range: 0.4-2.3 g/L). Serum free light chain test revealed 284.48 mg/L (normal range: 3.3-19.5 mg/L) and 23.1 mg/L (normal range: 5.71-26.3 mg/L) of kappa and lambda, respectively, with an increased ratio of 12.32 (normal range: 0.26-1.65). Complement levels (C3, C4 and CH50) were normal. Hepatitis C antibody, autoimmunity, rheumatic factor, cryoglobulins, perinuclear antineutrophil cytoplasmic antibodies, serum anti-phospholipase A2 receptor (anti-PLA2R) antibodies were all negative. HBV-DNA viral load was 203 IU/mL (normal range< 100 IU/mL).

### 2.1 Kidney pathology and diagnosis

**Figure 1:**
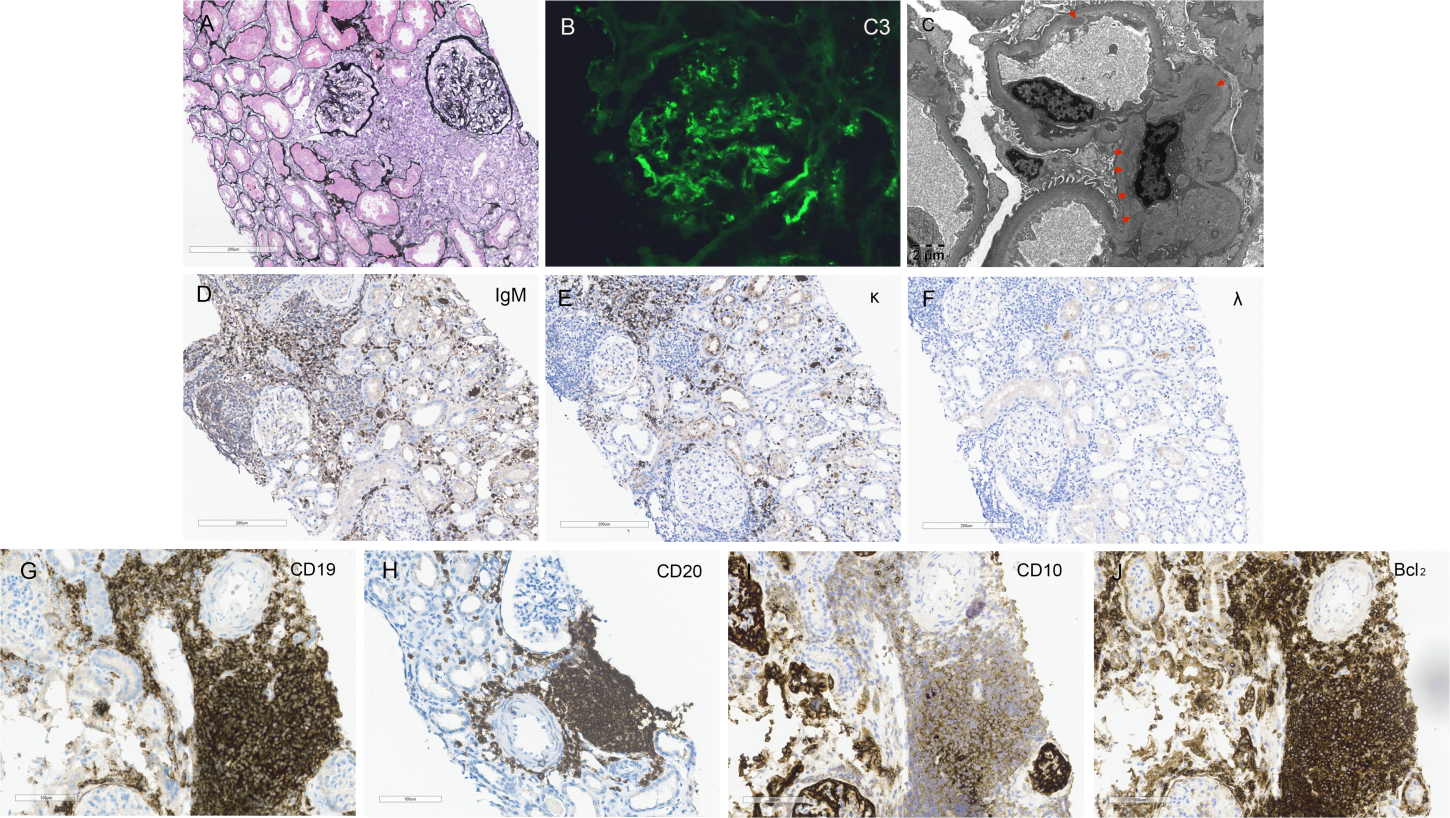
Kidney Histology and Immunohistochemistry Findings. **(A)** Light microscopy reveals a mesangial proliferative pattern of injury with multifocal infiltration of lymphoma cells in the interstitium (Periodic acid-silver methenamine staining, scale bar = 200 μm). **(B)** Immunofluorescence staining demonstrates bright C3 deposition along capillary walls and within mesangial areas. **(C)** Electron microscopy shows electron-dense deposits in mesangial and endothelial regions (red arrows, scale bar = 2 μm). **(D–F)** Immunohistochemistry staining within the areas of lymphoma cell infiltration in the renal interstitium is positive for IgM and kappa light chain and negative for lambda light chain (scale bar = 200 μm). **(G–J)** Immunohistochemistry staining for general lymphoma markers CD19 and CD20, and follicular lymphoma-specific markers CD10 and Bcl2, are all positive within the renal interstitium (scale bar = 200 μm).

A percutaneous renal biopsy was then performed. Of the 30 glomeruli sampled for light microscopy, 4 were severely found mesangial expansion and hypercellularity,18 were mild-to-moderate hypercellularity and 1 had endocapillary hypercellularity. There was focal acute tubular injury, erythrocyte casts, mild tubular atrophy and interstitial fibrosis. Multifocal lymphoid cell-like aggregation was found in renal interstitium (**Figure 1A**).

In the glomerular mesangial and capillary wall regions, immunofluorescence revealed diffuse C3 (2+) deposition (**Figure 1B**). Staining for IgA, IgG, IgM, C1q, and HBV antigens was negative in both frozen and enzyme-digested sections. In the renal interstitium, where lymphoma cells were infiltrating, immunohistochemistry showed positive staining for IgM-kappa light chain but negative for lambda light chain (**Figures 1D–F**). Electron microscopy demonstrated mesangial cell and matrix proliferation with electron-dense deposits in the mesangial areas and extensive podocyte foot process fusion (**Figure 1C**). Immunohistochemistry for general lymphoma markers (CD19 and CD20) and follicular lymphoma-specific markers (CD10 and Bcl2) was positive (**Figures 1G–J**), while Congo Red staining was negative. The renal pathology diagnosis is as follows: (1) The findings are consistent with follicular lymphoma involving the kidneys, in accordance with the patient’s history and immunophenotyping results. (2) The findings are consistent with mesangial proliferative glomerulonephritis. C3 glomerulopathy cannot be excluded, and follow-up on complement C3 levels is recommended. (3) Acute tubular injury with repair is present. Based on the patient’s medical history and renal pathology, and in conjunction with the detection of M protein in the serum by immunofixation electrophoresis, the case is diagnosed as renal involvement secondary to follicular lymphoma, including MIg-C3GN, lymphoma interstitial infiltration and acute tubular injury.

To complete the diagnostic workup, the patient was evaluated for abnormalities in the alternative complement pathway. Blood samples were sent to a specialized third-party laboratory (KingMed Diagnostics) for comprehensive analysis of complement-related antibodies and genetic mutations. The results showed positive autoantibodies against complement C3 convertase and complement factor H. Levels of complement factors CFH, CFB, and CFI were within the normal range. No genetic variants or mutations were identified in the CFH, CFB, or CFI genes.

### 2.2 Treatment and follow-up

#### 2.2.1 Hematological response

After discharge from the nephrology department, the patient has been undergoing treatment and follow-up in the hematology department. Starting in March 2023, the patient received three cycles of G-CHOP chemotherapy. Following the second cycle, the patient achieved partial response (PR). However, the patient subsequently contracted SARS-CoV-2 infection and developed herpes zoster on the skin. After the third cycle of G-CHOP chemotherapy, the therapeutic efficacy was poor, and there was a suspicion of transformation into diffuse large B-cell lymphoma (DLBCL). Therefore, Polatuzumab (Pola, a CD79b monoclonal antibody) was added to the regimen for the fourth cycle. Unfortunately, the patient’s disease progressed (PD) and was deemed refractory follicular lymphoma.

Subsequently, the patient’s regimen was changed to Obinutuzumab, Zanubrutinib, and Lenalidomide. After two cycles, stable disease (SD) was achieved and treatment continued to the sixth cycle. At this point, the M spike was undetectable, the serum kappa/lambda free light chain ratio was 2.306 (kappa 20.8 mg/L, lambda 9.02 mg/L), and a follow-up PET-CT showed reduced lymph node size and metabolic activity. However, new hepatic nodules were detected, but biopsy was not feasible due to their small size. Efficacy could not be definitively evaluated, so the patient continued maintenance therapy with the same regimen for a planned 18 months. However, the patient discontinued treatment on their own. In March 2024, a PET-CT indicated disease recurrence and progression. The patient then received Linperlisib at another hospital, but the disease continued to progress. In November 2024, the patient returned to our hospital and the regimen was changed to Obinutuzumab, a PD-1 monoclonal antibody, and Chidamide. The efficacy of this new regimen is yet to be evaluated. The patient has not yet returned to the hospital for follow-up.

#### 2.2.2 Renal outcomes

**Figure 2:**
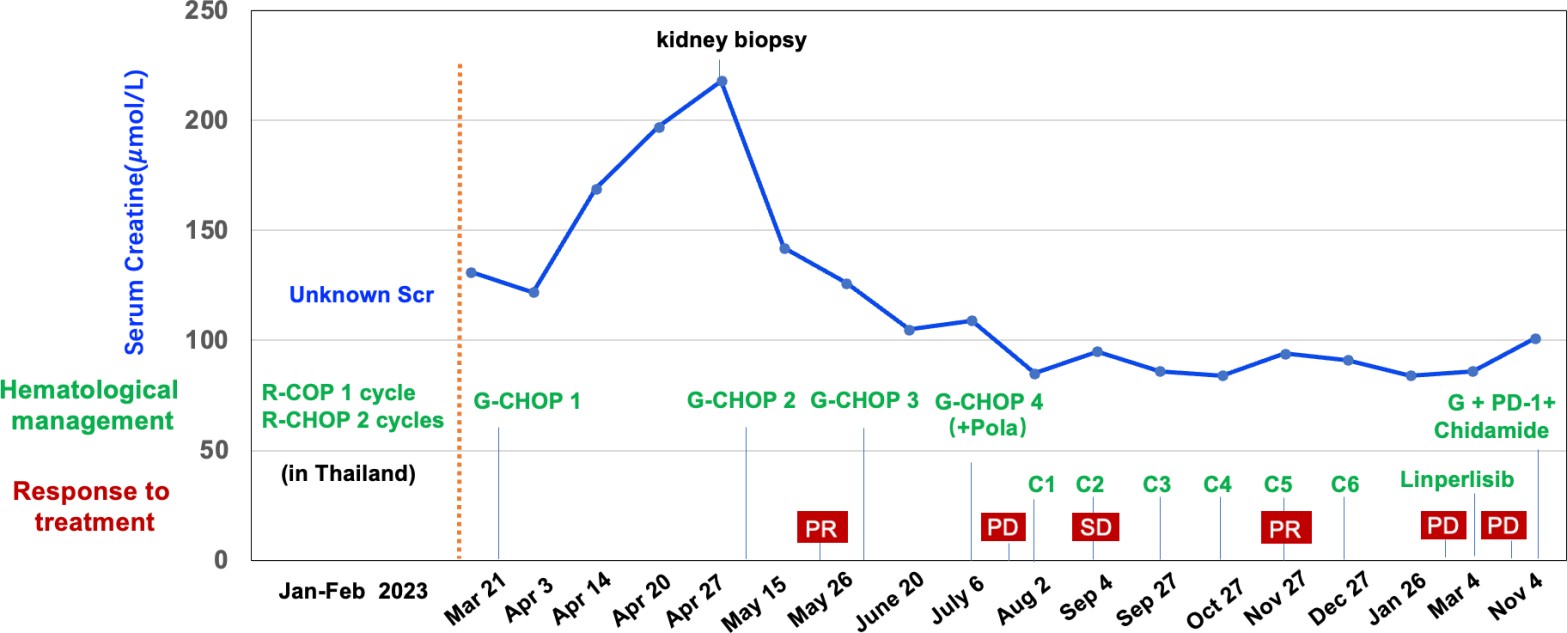
Clinical course, renal function, hematological management and response to the treatments of the case. R, Rituximab; C, Cyclophosphamide; O, Vincristine; P, Prednisone; H, Epirubicin; G, Obinutuzumab; Pola, Polatuzumab; C1-C6, Obinutuzumab + Zanubrutinib + Lenalidomide; PR, partial response; PD, progressive disease; SD, stable disease.

Following the second cycle of G-CHOP chemotherapy, the patient’s spot urinary total protein-to-creatinine ratio improved to 0.189 g/g, and subsequent urinalysis showed negative proteinuria. Renal function normalized and remained stable throughout the follow-up period (**Figure 2**). Additionally, HBV-DNA levels were consistently suppressed below 100 IU/mL during the entire follow-up period.

## 3 Discussion

C3GN is a rare glomerular disease associated with dysregulation of the alternative pathway (AP) of the complement system, characterized by predominate C3 deposits with little immunoglobulins deposition in the kidney (4–6). In this case, kidney immunofluorescence revealed bright C3 deposits in the glomeruli. We initially performed paraffin immunofluorescence to detect possible masked immunoglobulin or monoclonal immunoglobulin (MIg) deposits, thereby avoiding a missed diagnosis of immune complex-mediated glomerulonephritis or proliferative glomerulonephritis with MIg deposits (PGNMID). The differential diagnosis of isolated C3 staining mainly includes C3 glomerulopathy and post-infectious glomerulonephritis (PIGN). Additionally, this lymphoma patient’s renal injury was accompanied by hepatosplenomegaly, a mild increase in serum IgM levels, and a monoclonal IgM kappa M-spike, suggesting another differential diagnosis of renal involvement associated with Waldenstrom’s macroglobulinemia (WM). However, the patient displayed normal serum complement levels, an insufficiently high serum IgM level, and kidney biopsy and bone marrow examination were not consistent with the diagnosis of WM.

Regarding the kidney dysfunction in this case, the extensive infiltration by lymphoma cells, as evidenced by renal pathology, coupled with the focal acute tubular injury, implies a multifaceted etiology for the increased serum creatinine levels. Lymphocyte infiltration appears to be the primary factor contributing to the elevation in serum creatinine, a hypothesis corroborated by the notable recovery of renal function post-treatment targeting the B-cell clone. Although C3 deposits were identified within the renal tissue, the absence of marked glomerular hypercellularity suggests that these deposits may not have been the primary driver of AKI. This multifactorial nature could also explain the relatively low level of proteinuria, which was predominantly non-albumin in nature.

Emerging data suggest the frequent presence of MIg in the serum of C3GN patients (7–10). Prevalence of MIg among C3GN-diagnosed patients ranges from 15 to 40%, and it is often associated with older age (≥ 50 years of age) and a heightened risk of kidney disease progression (10). Case series have cumulatively described more than 150 patients with MIg-C3GN such as monoclonal gammopathy of renal significance (MGRS, 60%-72.2%), multiple myeloma (MM,19.5%-34%) and chronic lymphocytic leukemia (CLL, 2.8%-6%). To our knowledge, only one case of lymphoma-associated MIg-C3G has been reported (in Sethi’s cohort, 2.8%) (10).

Follicular lymphoma is a common type of non-Hodgkin lymphoma, but its association with MIg-C3GN is rare, thereby making our case an unusual instance of paraneoplastic glomerular disease. Light microscopy findings in our case showed not only the mesangial proliferative pattern injury of C3GN, but also multifocal lymphoma cell infiltration in the interstitium, unlike the previous reported cases with either pure C3GN or concurrent thrombotic microangiopathy (TMA) or light-chain proximal tubulopathy (LCPT). Therefore, the unique aspect of renal involvement in this case of follicular lymphoma lies in the dual mechanisms of monoclonal immunoglobulin-mediated injury: indirect damage through C3 glomerulonephritis and direct involvement via lymphoma cell infiltration into the renal interstitium with secretion of monoclonal IgM-kappa light chains.

The pathogenesis of C3GN is due to dysregulation of complement AP activation. This dysregulation can manifest as the presence of C3 nephritic factor (C3NeF), antibodies against complement factor H (CFH), gene variants/mutations affecting CFH or complement factor B (CFB) (4–6). Monoclonal immunoglobulins (MIg) may also contribute to C3GN pathogenesis by disrupting the regulation of the AP. In the initial report by Zhang et al. on 19 Chinese MIg-C3GN patients, complement-related tests, including CFH, anti-CFH antibody, and C3NeF, were conducted in 12 patients (8). Three patients had deceased CFH levels with synchronous low C3 levels. Only one patient showed positive anti-CFH antibody and C3NeF. In Sethi’s report about 26 patients with MIg-C3GN who were evaluated for abnormalities of the alternative pathway of complement, 11 patients had C3NeF, 2 patients with autoantibodies to CFH and one to CFB (10).

CFH is a critical regulator of AP, both in the fluid phase (blood) and on the surfaces of cells. Mutations or polymorphisms that impair CFH’s ability to recognize host cell surfaces can lead to complement-mediated tissue damage and disease. There is evidence that MIg may inhibit regulation of the AP by acting as C3 nephritic factor or by interfering with CFH. In a MIg-C3GN case who had IgG-kappa monoclonal gammopathy, the monoclonal protein behaved as a factor H inhibitor (11).

In the present case, we describe a patient with lymphoma and MIg-C3GN who exhibited both a monoclonal IgM kappa M-spike and autoantibodies targeting complement C3 convertase and complement factor H (CFH). While minimal change disease is the most frequent glomerular pathology observed in lymphoma patients, lymphoma-associated MIg-C3GN is exceptionally rare. To date, only two publications have reported a total of four cases of MIg-C3GN with monoclonal IgM (7, 10), but the etiology of these cases is unclear. Additionally, a cohort study involving 382 patients with lymphoid neoplasms and an IgM M protein demonstrated a 4.7% prevalence of follicular lymphoma (12). In our case, the positive immunohistochemical staining for IgM-kappa light chains within the interstitial lymphoma strongly suggests that the follicular lymphoma is the source of the monoclonal IgM-kappa. Drawing on the mechanisms described in other types of monoclonal immunoglobulin-associated C3GN (8, 13), we speculate that IgM-kappa free light chains, derived from the lymphoma, may function as mini-autoantibodies targeting the N-terminus of CFH, thereby disrupting its cofactor activity, overactivating the alternative pathway of the complement system and inhibiting the function of complement regulatory proteins. This leads to excessive C3 deposition in the glomeruli, resulting in immunoglobulin-negative, C3-positive glomerulonephritis.

The concurrent presence of these autoantibodies and a monoclonal IgM kappa M-spike in MIg-C3GN has not been previously reported. However, a noteworthy example is a 76-year-old patient with MIg-C3GN reported from China, in whom the anti-CFH autoantibodies purified from the patient’s plasma exchange fluids were proven to be a monoclonal IgGλ, indicating that monoclonal antibodies may interact with multiple components of the complement cascade (14). Further research is necessary to investigate the mechanisms by which monoclonal antibodies produced by lymphoma contribute to complement dysregulation in patients with MIg-C3GN.

Treatment strategies for MIg-C3GN remain uncertain and have primarily focused on supportive measures, immunosuppression, or plasmapheresis, with inconsistent and varied efficacy (15). Among 19 reported cases of MIg-C3GN in China, 3 patients died (15.8%), indicating poor renal outcomes under current immunosuppressive or conservative therapies. Immunosuppressant therapies show no advantage over supportive therapy in terms of renal prognosis, and the benefit of clone-targeted chemotherapy is still under investigation (8). In contrast, a French study reported a mortality rate of 10%. Case series and reports of patients with MIg-C3GN treated with myeloma-directed therapies have shown promising renal response rates. Patients who achieved hematological response after chemotherapy had higher renal response rates and median renal survival than those receiving conservative or immunosuppressive therapy (9). Anti-CD38 therapy with daratumumab may prevent renal progression in the setting of MIg-C3GN (16). However, there is no published literature documenting experience with follicular lymphoma-associated C3GN. In this case, the patient successively underwent various chemotherapy and targeted therapy regimens. During nearly 2 years of follow-up, the patient’s renal outcomes have been favorable, with hematuria and proteinuria resolved and renal function normalized and maintained. This indicates that lymphoma-directed therapy can improve renal outcomes in patients with MIg-C3GN. In terms of hematological response, the patient achieved partial remission at one point, but the lymphoma later relapsed and progressed. Treatment was deemed ineffective, with suspicion of transformation into diffuse large B-cell lymphoma.

The patient’s concurrent hepatitis B virus (HBV) infection adds complexity to the case. Previous evidence has suggested that hepatitis viruses, such as HBV and HCV, have a certain association with non-Hodgkin’s lymphoma (NHL) (17). In this patient, HBV infection may have contributed to the lymphomagenesis of follicular lymphoma, resulting in the production of monoclonal IgM-kappa, which led to MIg-C3GN. Regarding treatment, continued antiviral therapy for HBV is essential to prevent viral replication and liver damage, particularly during immunosuppression from chemotherapy for follicular lymphoma. The requirement to maintain anti-HBV therapy concurrently with chemotherapy emphasizes the need for a multidisciplinary approach to manage the patient’s complex medical conditions effectively.

## 4 Conclusion

In lymphoma patients, the most common type of glomerular injury is minimal change disease, while lymphoma-associated MIg-C3GN is extremely rare. We report the first case of MIg-C3GN secondary to follicular lymphoma. The uniqueness of this case lies in the diverse renal pathological manifestations: not only is there indirect renal damage caused by M protein, but there is also direct injury to the renal interstitium by M protein, accompanied by acute tubular injury. These findings have not been previously documented in MIg-C3GN patients. Although the treatment for MIg-C3GN remains uncertain, therapies targeting lymphoma may help improve renal outcomes.

## Data Availability

The raw data supporting the conclusions of this article will be made available by the authors, without undue reservation.
