# Supplementary figures and images for: Monoclonal gammopathy-associated C3 glomerulonephritis secondary to follicular lymphoma: a case report
Source: Front Immunol. 2025 Apr 24;16:1551788. doi: 10.3389/fimmu.2025.1551788 (PMC12058688; doi:10.3389/fimmu.2025.1551788)

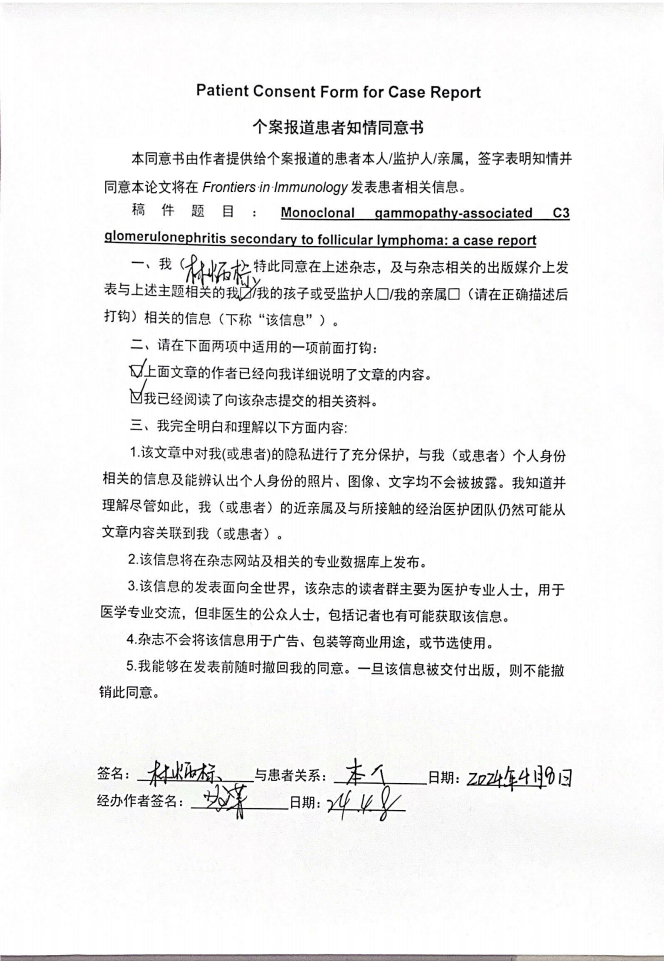

Supplement: Supplementary file 2 [file Image1.png]
